# Comprehensive Risk Assessment of LAD Disease Progression in CCTA: The CLAP Score Study

**DOI:** 10.3390/jcdd11110338

**Published:** 2024-10-23

**Authors:** Antonella Tommasino, Federico Dell’Aquila, Marco Redivo, Luca Pittorino, Giulia Mattaroccia, Federica Tempestini, Stefano Santucci, Matteo Casenghi, Francesca Giovannelli, Stefano Rigattieri, Andrea Berni, Emanuele Barbato

**Affiliations:** 1 Division of Cardiology, Sant’Andrea Hospital, Via di Grottarossa 1035, 00189 Rome, Italy; federico.dellaquila00@gmail.com (F.D.); marco.redivo95@gmail.com (M.R.); luca.pittorino@gmail.com (L.P.); giuliamattaroccia@yahoo.it (G.M.); federicatempestini95@gmail.com (F.T.); 62santucci@gmail.com (S.S.); matcasenghi@hotmail.it (M.C.); francy_giovannelli@yahoo.it (F.G.); stefanorigattieri@yahoo.it (S.R.); andrea.berni@uniroma1.it (A.B.); emanuele.barbato@uniroma1.it (E.B.); 2 Department of Clinical and Molecular Medicine, Sapienza University of Rome, Via di Grottarossa 1035, 00189 Rome, Italy

**Keywords:** coronary artery disease, left main bifurcation angle, atherosclerotic plaque development, risk stratification

## Abstract

Background: a wider left main bifurcation angle (LMBA) has been linked to severe plaque development in the proximal left anterior descending artery (LAD). This study aimed to identify predictors of severe proximal LAD stenosis and major adverse cardiovascular events (MACE) using coronary computed tomography angiography (CCTA). Methods: from an initial cohort of 650 consecutive patients, we analyzed 499 patients who met the inclusion criteria after exclusions. Plaque morphology and characteristics were assessed by CCTA, and MACE occurrences were recorded at follow-up. A predictive score for LAD disease progression (CLAP score) was developed and validated. Results: severe proximal LAD stenosis was detected in 32% (160/499) of patients by CCTA. MACE occurred in 12.5% of patients at follow-up. Significant predictors of MACE were LMBA > 80° (HR: 4.47; 95% CI: 3.80–6.70; *p* < 0.001), diabetes (HR: 2.94; 95% CI: 1.54–4.63; *p* = 0.031), chronic kidney disease (HR: 1.71; 95% CI: 1.31–6.72; *p* = 0.041), high-risk plaques (HR: 2.30; 95% CI: 1.45–3.64; *p* < 0.01), obstructive CAD (HR: 2.50; 95% CI: 1.50 to 4.10, *p* = 0.01), and calcium score (CAC) (HR: 1.05; 95% CI: 1.02–1.08, *p* = 0.004). The CLAP score demonstrated good discriminatory power in both the development (AUC 0.91; 95% CI: 0.86–0.96) and validation cohorts (AUC 0.85; 95% CI: 0.79–0.91); Conclusions: LMBA > 80°, diabetes, chronic kidney disease, obstructive CAD, CAC score >180 and high-risk plaques were significant predictors of MACE in CCTA patients. The CLAP score effectively predicted LAD disease progression, aiding in risk stratification and optimization of intervention strategies for suspected coronary artery disease.

## 1. Introduction

A wider left main bifurcation angle (LMBA) has been associated with severe plaque development in the proximal left anterior descending artery (LAD) [1,2]. The low and oscillatory flow turbulence at the bifurcation has been proposed as one of the possible mechanisms leading to endothelial dysfunction, an increase in the expression of pro-inflammatory and pro-atherogenic genes, and a promotion of lipoprotein retention in the arterial wall, which in turn favors plaque development [3,4].

Coronary computed tomography angiography (CCTA) has become a reliable non-invasive imaging technique for the assessment of coronary artery disease (CAD) [5,6]. However, the role of CCTA-derived LMBA, in conjunction with plaque characteristics, in predicting proximal LAD stenosis and major adverse cardiac events (MACE) remains unclear. Although the association between LMBA and the development of atherosclerotic lesions in the proximal LAD has been demonstrated, the prognostic value of this parameter in relation to plaque characteristics has not yet been fully explored. Furthermore, there is a lack of validated risk stratification tools that incorporate both clinical and anatomical factors to guide personalized treatment strategies in patients undergoing CCTA. This study aims to address these knowledge gaps and evaluate the potential clinical implications of LMBA and plaque features in the context of risk stratification and treatment planning.

## 2. Materials and Methods

### 2.1. Study Design and Patient Selection

This single-center observational registry was conducted in compliance with the principles of the Declaration of Helsinki and the relevant local regulations. Consecutive patients undergoing CCTA for suspected CAD between March 2021 and March 2023 were included in the study. The inclusion criteria were as follows: (1) age ≥ 18 years; (2) referral for CCTA due to chest pain or other symptoms suggestive of CAD. Patients were excluded if they (1) had poor image quality on CCTA; (2) had a history of coronary artery bypass grafting (CABG); (3) had a ramus intermedius; (4) had a glomerular filtration rate < 30 mL/min/1.73 m^2^ or were on dialysis.

Demographic and clinical data were collected. The degree of luminal stenosis was assessed to determine the severity of the disease. In a subset of patients undergoing invasive coronary angiography (ICA), LMBA was also analyzed using 3D-QCA software (CardiOp-B system, version 2.1.0.151, Paieon Medical Ltd., Rosh Ha’ayin, Israel). The LMBA was defined as the angle between the left main coronary artery, the proximal LAD and the proximal left circumflex artery (LCX).

### 2.2. CCTA Acquisition and Analysis

CCTA scans were performed according to a standardized protocol using a Revolution CT Evo 128-slice scanner (GE Healthcare, Chicago, IL, USA). All patients received sublingual nitroglycerin (0.4 mg) and beta-blockers (intravenous metoprolol 5–30 mg) if their heart rate was at least 60 beats per minute unless contraindications were present. After a standard supine examination, a non-contrast scan was performed to assess coronary artery calcification. This consisted of a single inspiratory breath-hold acquisition in the craniocaudal direction, extending from the carina to the inferior border of the heart. Contrast-enhanced angiography was then performed, timing the arrival of the contrast agent (iopamidol 370, 76%; Bracco Group, Milan, Italy) with a threshold of 100 Hounsfield units in a region of interest in the ascending aorta, using the bolus-tracking technique. A contrast bolus of 50 to 70 mL, followed by 50 mL of saline, was injected at a rate of 4–5 mL/s through a peripheral vein.

All datasets from the CCTA scans were transferred to a separate workstation equipped with Volume Viewer software version 10.3.67.ar (GE Healthcare, Chicago, IL, USA) for image postprocessing and analysis. LMBA measurements were obtained using 2D and 3D volume reconstructions. Two experienced operators independently analyzed the images to ensure accuracy and interobserver reliability. In addition to the assessment of luminal stenosis and LMBA, CCTA datasets were further analyzed to characterize plaque morphology. Plaque morphology was evaluated using a standardized protocol [7]. Plaques were classified as calcified, partially calcified or non-calcified based on their attenuation values. The presence of high-risk plaque features, such as positive remodeling, low attenuation plaque, napkin ring sign, and spotty calcifications was also recorded [8]. Coronary calcifications were quantified using the Agatston method [9]. The total calcium score was calculated for each patient, and the presence of calcifications in the proximal LAD was specifically noted.

The distance from the left main bifurcation to the origin of the proximal LAD plaque was measured using multiplanar reconstructions. Coronary tortuosity was assessed using a 3D tortuosity index defined as the ratio of the actual vessel length to the straight-line distance between the proximal and distal endpoints as the presence of a bend greater than 90° or three bends from 45° to 90° within the lesion segment [10].

### 2.3. Invasive Coronary Angiography and 3D-QCA Image Acquisition and Analysis

Coronary angiography was performed using standard techniques and a minimum frame rate of 15 frames per second. Three-dimensional coronary artery reconstructions were performed offline using validated software (CardiOp-B system, version 2.1.0.151, Paieon Medical Ltd., Rosh Ha’ayin, Israel) by an experienced operator blinded to individual patient data and clinical outcomes. The protocol for 3D reconstruction has been described in detail elsewhere [11,12,13]. In brief, two orthogonal angiographic views were analyzed, showing the minimal overlap of the distal vessel and the side branch (SB), separated by an angle of at least 30 degrees. In the left main branch (LM), the left anterior descending coronary artery (LAD) was designated as the distal main vessel (DMV) and the left circumflex (LCX) as the SB. Significant stenosis in the proximal LAD was defined as a reduction in luminal diameter of 70% or more.

### 2.4. Follow-Up and Definitions

One-year clinical follow-up was carried out by direct telephone contact or during visits to the outpatient clinic. Disease progression in the proximal LAD was assessed through follow-up imaging (ICA or CCTA), which was performed when clinically indicated or in cases where baseline CCTA or ICA had identified a plaque with high-risk features, even if not significant. Disease progression was defined as an increase in luminal stenosis of at least 30% from baseline, as well as the appearance of new high-risk plaque features, such as positive remodeling, low attenuation plaque, napkin ring sign, or new calcifications in the proximal LAD segment. To determine disease progression, patients with discrepancies between CCTA and subsequent ICA results were considered based on the criterion that ICA is the gold standard for anatomic assessment. If ICA showed a higher degree of stenosis than CCTA, this was considered as progression of disease. This was done to ensure consistency in the assessment of changes in severity of stenosis.

TLR was defined as repeated PCI treatment within the target lesion stent or within 5 mm of the stent edge.

*Primary endpoints:*

(a) To evaluate the consistency and predictive value of LMBA measurements in determining the risk of significant stenosis in the proximal LAD when measured by CCTA and 3D-QCA.
(b) To assess the incidence of major adverse cardiac events (MACE), defined as a composite of cardiovascular death (CD), myocardial infarction (MI), percutaneous coronary intervention (PCI), target lesion revascularization (TLR), and progression of atherosclerotic disease at the proximal LAD at follow-up.

*Secondary endpoints:*

(a) Determination of the individual components of MACE, in particular the progression of atherosclerotic disease at the proximal LAD and the occurrence of target lesion revascularization (TLR);
(b) Identification of the clinical and anatomical predictors of proximal LAD stenosis.

**Statistical Analysis**

Continuous variables were expressed as mean ± standard deviation or median (interquartile range) and compared using Student’s *t*-test or Mann-Whitney U test, as appropriate. Categorical variables were expressed as frequencies and percentages and compared using the chi-square test or Fisher’s exact test.

Univariate and multivariate logistic regression analyses were performed to identify predictors of severe stenosis in the proximal LAD. Variables with a *p*-value < 0.10 in the univariate analysis were included in the multivariate model. The Cox proportional hazards model was used to identify predictors of MACE, with results expressed as hazard ratios (HR) and 95% confidence intervals (CI).

To evaluate the diagnostic accuracy of CCTA and 3D-QCA for proximal LAD stenosis, an analysis of the receiver operating characteristic (ROC) curve was calculated. The area under the curve (AUC) was calculated and compared with the DeLong test. The Youden J index was used to determine the optimal LMBA value in terms of sensitivity and specificity. Bland–Altman plot analysis was used to estimate the mean difference in bifurcation angle measurements between CCTA and ICA.

To evaluate potential bias introduced by the inclusion of patients with prior PCI, a sensitivity analysis was performed by excluding patients who had undergone PCI, particularly in the LAD. This analysis aimed to determine whether the exclusion of these high-risk patients would affect the predictive power of the model. The hazard ratios (HRs) for each predictor were recalculated, and the area under the ROC curve (AUC) was compared between the models with and without patients with prior PCI, using the DeLong test.

Based on the results of our multivariate analyses, we developed a composite score that stratifies the risk of disease progression in the LAD in patients undergoing CCTA. The score was calibrated according to the TRIPOD statement [14], and its predictive capacity was assessed using the Brier score. The final score models were subjected to internal validation using the bootstrapping out-of-bag estimation method.

The performance of the scores was assessed using the area under the ROC curve (AUC), calibration (Hosmer-Lemeshow test) and discrimination ability (Net Reclassification Improvement). The external validation was performed on an independent cohort of 200 patients who had undergone CCTA. These patients were recruited from an external hospital and spanned the period from March 2022 to March 2023. All patients met the inclusion criteria of a CCTA for coronary artery disease assessment and were followed in a similar manner to the development cohort.

The sample size was determined based on the expected incidence of MACE in patients undergoing CCTA for suspected CAD. Previous studies in similar populations have reported MACE incidence rates of approximately 10% to 15% [15]. To ensure adequate power to detect a significant difference in outcomes between patients with and without significant proximal LAD stenosis, a power analysis was performed. Assuming a hazard ratio of 2.0 for MACE, a two-sided alpha of 0.05, and a power of 80%, it was calculated that a minimum of 450 patients would be required to detect a statistically significant difference in MACE rates.

All statistical analyses were conducted using IBM SPSS Version 29.0.1.0. A *p*-value of less than 0.05 was considered statistically significant.

## 3. Results

A total of 650 consecutive patients who underwent CCTA were initially included in the study. Sixty-six (10.2%) patients were excluded due to poor image quality or a history of coronary artery bypass grafting (CABG). In addition, eighty-five (13.1%) patients were found to have a ramus intermedius and were also excluded from the analysis to avoid anatomic confounding factors when analyzing LMBA. The final study population consisted of 499 patients, whose main baseline clinical and anatomical characteristics are listed in Table 1.

Of the final cohort of 499 patients, 288 (57.7%) underwent subsequent ICA. In this group, CCTA revealed significant stenosis in the proximal LAD artery in 204 patients (70.8%). This finding was confirmed by ICA in 124 patients (60.8% of those with significant stenosis detected by CCTA, or 43.1% of the ICA group). Additionally, 80 patients (27.7% of the ICA group) were found to have moderate stenosis. Percutaneous coronary intervention (PCI) on the proximal LAD was performed in 110 cases (38.2% of those who underwent ICA), while 14 patients were referred for surgery due to significant distal LM disease.

Plaque morphology assessment at CCTA revealed that patients with significant proximal LAD stenosis had a higher prevalence of calcified plaques (45.2% vs. 28.6%, *p* < 0.001). The median calcium score was higher in patients with significant proximal LAD stenosis (180 (IQR: 55–480) vs. 75 (IQR: 15–250), *p* < 0.001). The presence of high-risk plaques, such as positive remodeling and low-attenuation plaque, was also more common in patients with significant stenosis (35.6% vs. 15.4%, *p* < 0.001). The mean LMBA was significantly wider in patients with significant LAD stenosis (106.4° ± 29.4°) than in patients without stenosis (74.7° ± 27.2°, *p* < 0.001). The distribution of the LMBA angle is shown in Figure S1.

The distance from the LM bifurcation (LMB) to the beginning of the proximal LAD plaque was shorter in patients with severe LAD stenosis than in those without stenosis (8.5 ± 4.2 mm vs. 12.3 ± 5.6 mm, *p* < 0.001) and showed a higher prevalence of vessel tortuosity (12.1% vs. 3.7%; *p* = 0.002). In addition, higher LMBA values (LMBA > 80°) were associated with both increased coronary tortuosity and a shorter distance between the LMB and the proximal LAD plaque. Specifically, patients with LMBA > 80° had a mean distance of 9.2 ± 1.2 mm from the LMB to the proximal LAD plaque, compared with 12.1 ± 2.3 mm in patients with LMBA ≤ 80° (*p* = 0.01). In addition, the prevalence of coronary tortuosity was significantly higher in patients with LMBA > 80° (13.9% vs. 5.2%, *p* = 0.02). Patients with higher bifurcation angles (LMBA > 80°) had significantly shorter LM lengths compared to patients with lower bifurcation angles. The mean LM length in patients with LMBA > 80° was 12.9 ± 2.3 mm, compared to 15.2 ± 2.5 mm in patients with LMBA ≤ 80° (*p* = 0.02).

### 3.1. Primary Endpoints

- Consistency and predictive value of LMBA measurements in CCTA and 3D-QCA modalities

**Figure 1:**
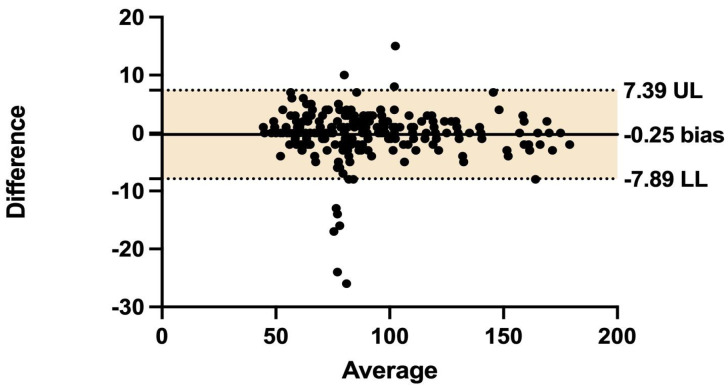
Difference vs. Average: Bland-Altman analysis of LMBA measurements from CCTA and 3D-QCA.

**Figure 2:**
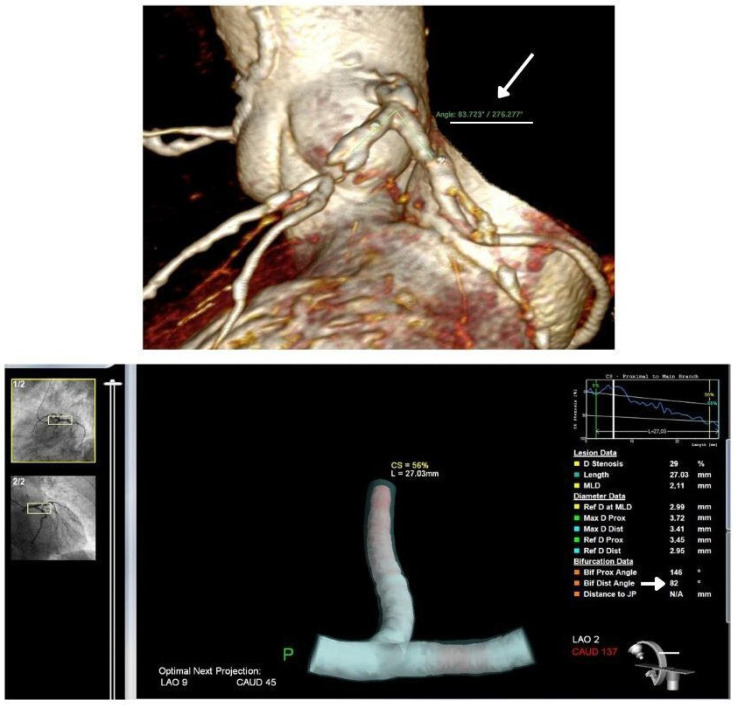
The left main bifurcation angle (LMBA) was measured on CTA (**top panel**) and on coronary angiographic images using 3D-QCA (**bottom panel**).

An insignificant discrepancy was found when comparing LMBA measurements between CCTA and 3D-QCA, with mean values of 88.73° and 88.98°, respectively. Bland-Altman analysis revealed an insignificant mean difference of −0.25° (95% CI: −7.89 to 7.39) (Figure 1 and Figure 2). The AUC for predicting significant proximal LAD disease was 0.84 (SE = 0.023, 95% CI: 0.796–0.890) for CCTA and 0.86 (SE = 0.022, 95% CI: 0.817–0.905) for 3D-QCA. The difference in AUC values between the modalities was −0.018 (SE: 0.0046, 95% CI: −0.0271 to −0.0090, *p* = 0.00009) (Figure S2). The optimal LMBA threshold for predicting significant LAD disease, balancing sensitivity and specificity, was identified at 80° (sensitivity: 0.93, specificity: 0.70).

- Clinical outcome and predictors of MACE
  a. Individual Counts for Each Endpoint.

**Figure 3:**
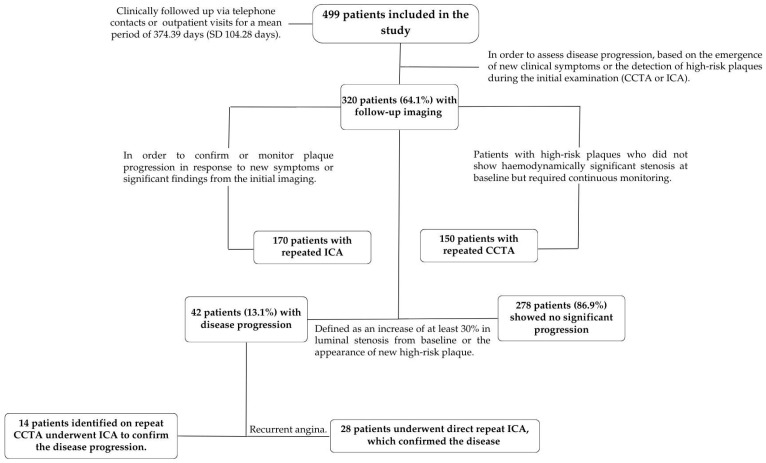
Flowchart for patient enrollment in the study and follow-up.

At a mean follow-up of 374.39 days (SD 104.28 days), MACE occurred in 12.47% of the patient population. Specific outcomes included TLR in 12 cases (2.4%), disease progression in the proximal LAD in 42 cases (8.4%), MI in a different territory in 5 cases (1%), and cardiovascular death in 3 cases (0.6%). Events were more frequently observed in patients with an LMBA > 80° compared to those with an LMBA ≤ 80° (Figure 3 and Table S1).

b. Predictors of MACE

The Cox proportional hazards model identified several significant predictors of MACE. Due to the significant collinearity between the CAC score and the presence of calcified plaques (VIF = 5.2), only the CAC score was included in the final model.

Diabetes was associated with a higher risk of MACE (HR: 2.94; 95% CI: 1.54 to 4.63, *p* = 0.031), as was chronic kidney disease (HR: 1.71; 95% CI: 1.31 to 6.72, *p* = 0.041). High-risk plaque morphology (HR: 2.30; 95% CI: 1.45 to 3.64, *p* < 0.01) and LMBA >80° (HR: 4.47; 95% CI: 3.80 to 6.70, *p* < 0.001) were also significant predictors of increased MACE risk (Table 2 and Figure 2). Obstructive CAD and CAC scores were also associated with an increased risk of MACE (HR: 2.50; 95% CI: 1.50 to 4.10, *p* = 0.01; HR: 1.05; 95% CI: 1.02–1.08, *p* = 0.004, respectively).

### 3.2. Secondary Endpoints

- Detailed Analysis of Predictors for Individual MACE Components

In the analysis of the individual components of MACE, LMBA > 80° was the strongest predictor of disease progression in the proximal LAD (HR: 5.325; 95% CI: 4.029–6.701, *p* < 0.001), followed by obstructive CAD (HR: 2.20; 95% CI: 1.40–3.50, *p* = 0.02), CAC score > 180 (HR: 1.04; 95% CI: 1.01–1.07, *p* = 0.01) and high-risk plaque (HR: 2.120; 95% CI: 1.385–3.246, *p* < 0.001). Additionally, diabetes was strongly associated with both TLR (HR: 4.683; 95% CI: 2.346–5.962, *p* < 0.001) and disease progression in the proximal LAD (HR: 3.745; 95% CI: 2.691–4.713, *p* < 0.001) (Table 3).

- Clinical and anatomical predictors of proximal LAD stenosis

Logistic regression analysis identified several variables that were significantly associated with severe stenosis in the proximal LAD. These included hypertension (OR: 1.228; 95% CI: 1.102–2.024, *p* = 0.025), diabetes (OR: 7.437; 95% CI: 4.343–12.734, *p* < 0.001), chronic kidney disease (OR: 2.024; 95% CI: 1.074–3.811, *p* = 0.029), LMBA >80° measured by CCTA (OR: 3.234; 95% CI: 2.025–4.442, *p* < 0.001), high coronary tortuosity (OR: 2.954; 95% CI: 1.47–5.935, *p* = 0.002), obstructive CAD (OR: 2.80; 95% CI: 1.80–4.20, *p* < 0.01) and CAC score (OR: 1.03; 95% CI: 1.01–1.06, *p* = 0.005), and the presence of high-risk plaque (OR: 2.45; 95% CI: 1.50–4.01, *p* < 0.001) (Table S2).

### 3.3. Predictive Score for Disease Progression in the LAD

Based on the results of our study, we developed the CCTA-based LAD Atherosclerosis Progression (CLAP) Score to include additional significant predictors of disease progression. The CLAP score includes the following variables: LMBA > 80° (3 points), diabetes (2 points), obstructive CAD (2 points), presence of high-risk plaques (2 points), and CAC score > 180 (1 point) (Table S3).

The score demonstrated good discriminatory ability in both the development cohort (AUC 0.91; 95% CI: 0.86–0.96) and the external validation cohort (AUC 0.85; 95% CI: 0.79–0.91) (Figure S3). The Brier score for the CLAP score was 0.18 in the development cohort and 0.20 in the external validation cohort.

In our population, patients with higher CLAP scores were at greater risk of significant disease progression in the proximal LAD, as assessed by CCTA at one year (Figure 3).

To better assess the impact of these predictors, we divided our study population into two distinct cohorts. The first cohort focused on the impact of the LMBA angle on subsequent clinical events, such as MACE, and included the entire study population of 499 patients. The second cohort, consisting of 320 patients with follow-up imaging, was used to evaluate disease progression over time, specifically in the proximal LAD.

In the first cohort (499 patients), patients with LMBA > 80° showed a significantly higher incidence of MACE (19.8% vs. 7.4%, *p* = 0.016) compared to those with LMBA ≤ 80°. Specifically, PCI (12.4% vs. 3.4%, *p* = 0.002), TLR (4.5% vs. 1.0%, *p* = 0.04), and progression of disease in the proximal LAD (14.9% vs. 4.0%, *p* = 0.007) were all significantly more frequent in the group with LMBA > 80°. The CLAP score demonstrated strong predictive power for these outcomes, with higher scores correlating with increased event rates (Table S1).

**Figure 4:**
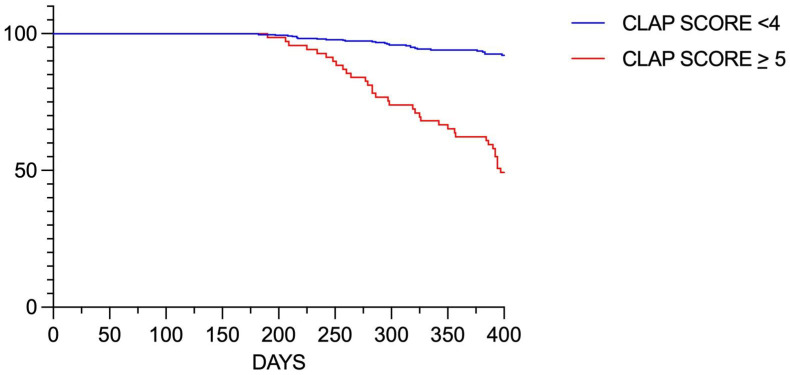
Survival curve showing the risk of significant disease progression in the proximal LAD as assessed by CCTA at one year.

In the second cohort (320 patients with follow-up imaging), 42 patients (13.1%) showed significant disease progression (Figure 4) The CLAP score was able to effectively predict disease progression, with an AUC of 0.89 (95% CI: 0.84–0.93) (Figure S5).

Patients with higher CLAP scores (6–10 points) had a markedly higher risk of disease progression compared to those with lower scores (0–2 points).

**Sensitivity analysis**

To assess the robustness of our results, a sensitivity analysis was performed excluding patients with previous PCI, especially those with previous LAD-PCI, to mitigate potential bias. The resulting cohort consisted of 389 patients. The major predictors of MACE, including LMBA > 80°, diabetes, high-risk plaque morphology, obstructive CAD, and CAC score, remained significant in the sensitivity analysis, with hazard ratios similar to those in the original model. Detailed results can be found in the Supplementary Materials. (Tables S5 and S6).

## 4. Discussion

Our study emphasizes the reliability of CCTA as a non-invasive imaging technique to assess coronary artery anatomy, especially its accuracy in measuring LMBA compared to 3D QCA. An LMBA of greater than 80 degrees was identified as the optimal threshold for predicting proximal LAD stenosis and total MACE [16,17,18,19,20,21,22,23]. Although in the subgroup analysis the difference for hard endpoints such as cardiovascular death or MI was not significant, this is likely due to the low number of these events in the overall study population, which may have limited the power to detect differences. Nonetheless, this study improves the understanding of LMBA’s role in CAD.

In line with previous research [24,25,26,27,28], our study confirms the strong association between diabetes and proximal LAD stenosis, underscoring the critical impact of metabolic dysregulation on coronary health. The increased risk of stenosis in patients with hypertension and chronic kidney disease further reinforces the well-established connection between systemic disease and coronary artery disease [29].

While standard risk factors are important for understanding atherosclerosis progression, they can fail to capture the complex mechanisms that drive CAD. This limitation highlights the need for more comprehensive risk assessment tools [30]. Many individuals who experience plaque rupture and myocardial infarction do not have standard modifiable risk factors, and most cardiovascular adverse events occur in patients with normal stress test results. This underscores the importance of detecting non-obstructive CAD to more effectively prevent MACE.

In addition to conventional risk factors, our study highlights the critical influence of anatomical factors like LMBA, coronary tortuosity, and high-risk plaques in predicting proximal LAD stenosis. The geometry of the left main bifurcation plays a crucial role in altering local hemodynamics, leading to endothelial dysfunction, lipid accumulation, and increased plaque vulnerability, which can result in adverse clinical events [5,31,32,33,34,35,36].

The role of vessel wall shear stress (WSS) in plaque formation and progression is also noteworthy. WSS represents the friction exerted by blood flow on the endothelial vessel wall. Variations in bifurcation angles and vessel curvature impact blood flow velocity, with high WSS typically associated with plaque rupture and low WSS associated with plaque progression and increased plaque burden [37,38,39]. Larger bifurcation angles contribute to lower WSS, which may promote plaque formation and progression at bifurcation sites [3,40]. These hemodynamic variations could lead to endothelial dysfunction and inflammation, critical drivers of atherosclerosis. Recognizing the relationship between bifurcation angles, WSS, and plaque vulnerability could enable the development of tailored imaging techniques or interventions to better detect and treat high-risk plaques.

Similarly, the increased risk of stenosis associated with shorter LM and coronary tortuosity underscores the importance of considering vessel geometry beyond the bifurcation region [33,37,41,42,43]. These findings suggest that a comprehensive approach, which considers both macroscopic arterial features and microscopic endothelial interactions, is essential for fully understanding atherogenesis.

Recently, there has been significant debate about the identification of high-risk plaques that, although not significantly obstructive, can develop rapidly and lead to acute events, as observed in our patient cohort [44,45]. A detailed analysis found that patients with high-risk plaques experienced faster disease progression, consistent with the study by Kinoshita et al., in which positive remodeling, low attenuation, napkin ring sign, and spotty calcification on CCTA were associated with OCT features of vulnerability [46].

In our analysis, obstructive CAD and CAC scores were also found to be independent predictors of MACE. The inclusion of CAC score allowed a quantitative assessment of coronary calcification, reflecting the overall burden of atherosclerosis. Our results are consistent with previous studies [47,48] that showed higher CAC scores correlated with an increased risk of adverse cardiovascular events. Assessing obstructive CAD, combined with plaque burden, highlights the need to consider both anatomic and functional disease characteristics in clinical decision-making [49].

To translate these findings into clinical practice, we developed the CLAP score, a novel tool to predict disease progression in the LAD. By combining clinical and anatomical factors, the CLAP score offers a comprehensive risk assessment. Its strong performance in both the development and validation cohorts underscores its potential to enhance personalized patient management and stratification. Although its predictive value for hard outcomes like CV death and MI should be validated in larger studies, the CLAP score provides a more targeted approach than traditional assessments, guiding personalized treatment strategies. Patients in the high-risk group may benefit from aggressive risk factor modification, closer monitoring, and earlier consideration of revascularization.

To further clarify the role of LMBA and the CLAP score in predicting clinical outcomes, we divided our population into two cohorts. In the first cohort, focused on clinical events, LMBA > 80° was associated with a significantly higher incidence of MACE and other events. In the second cohort with follow-up imaging, the CLAP score demonstrated strong predictive power for disease progression in the proximal LAD.

The sensitivity analysis, which excluded patients with previous PCI, showed that this had no significant impact on the hazard ratios of the primary predictors of MACE. This suggests that the predictive model and CLAP score remain robust, thus improving the generalizability of our results. Our findings align with previous studies demonstrating the additional prognostic value of plaque characteristics beyond traditional stenosis assessments [50,51,52,53,54].

For example, the ICONIC, SCOT-HEART, and PROMISE studies highlighted plaque composition and burden as strong predictors of future acute coronary syndromes, independent of stenosis severity [55,56,57]. However, while the CLAP score builds on these insights by incorporating high-risk and calcified plaques with the LMBA, its role as a predictor for major clinical outcomes like CV death and MI should be further explored in future studies. The integration of anatomical and clinical factors in the CLAP score highlights its potential utility in guiding personalized treatment strategies, especially in high-risk patients where traditional assessments may be insufficient.

- Study limitations

The retrospective design of the study may lead to selection bias and limit the generalizability of the results. Secondly, the study focused on anatomical parameters, while other factors, such as hemodynamic forces, were not considered. The number of events at follow-up in our study was relatively small, which limited the number of variables that could be included in the Cox regression analysis. Moreover, unlike the PARADIGM registry [58], the CLAP score does not include a direct measure of plaque progression. Although disease progression was assessed in selected patients through follow-up imaging (CCTA or ICA) when clinically indicated, or in cases where high-risk plaque features were present at baseline, a standardized imaging follow-up protocol for all patients was not conducted. However, the CLAP score does account for variables likely associated with a higher risk of progression, such as diabetes and the presence of high-risk plaques. We believe that a prospective study involving systematic follow-up imaging would help further validate the predictive power of the CLAP score.

## 5. Conclusions

In conclusion, our study demonstrates the reliability of LMBA measurements between CCTA and 3D-QCA and identifies important predictors of proximal LAD stenosis and total MACE. Although LMBA alone may not be sufficient to predict hard endpoints such as cardiovascular death or MI, it remains a valuable parameter for the assessment of proximal LAD stenosis and overall MACE. The introduction of the CLAP score provides a practical risk stratification tool that improves personalized treatment strategies by integrating clinical and anatomical factors. These results emphasize the importance of including anatomical parameters in risk assessment models and highlight the potential of CCTA as a non-invasive technique for comprehensive coronary artery assessment. Further studies are needed to validate these results for hard clinical outcomes.

## Figures and Tables

**Table 1:** Baseline characteristics of 499 patients included in the study.

Overall Population (n = 499)	Mean (SD) or Frequency
Age (yrs)	64.39 ± 9.9
Male	391 (78.3%)
Smoke	99 (19.8%)
Hypertension	365 (73.1%)
Dyslipidemia	280 (56.1%)
Diabetes Mellitus	113 (22.6%)
Chronic kidney disease	93 (18.6%)
1-vessel disease	252 (50.5%)
2-vessels disease	141 (28.3%)
3-vessels disease	96 (19.2%)
LM disease Proximal LAD disease	110 (22.1%) 210 (42.1%)
LAD PCI	110 (22.0%)

**Table 2:** Logistic regression analysis for predictors of MACE. The table shows the regression coefficients (B), standard errors (SE), Wald statistics, *p* values, odds ratios (Exp(B)), and 95% confidence intervals (CI) for the odds ratios of each variable.

Variable	B	SE	Wald	*p* Value	Exp (B)	95% LCI	95% UCI
Diabetes	1.077	0.281	14.72	0.031	2.938	1.540	4.627
Chronic kidney disease	0.537	0.422	1.62	0.041	1.709	1.310	6.720
CCTA LMBA > 80°	1.499	0.206	53.04	<0.001	4.474	3.799	6.701
High-risk Plaque	0.833	0.234	12.68	<0.01	2.298	1.449	3.644
CAC Score	0.048	0.017	8.15	0.004	1.05	1.02	1.08
Obstructive CAD	0.917	0.310	8.75	0.01	2.50	1.50	4.10

**Table 3:** Logistic regression analysis for predictors of disease progression int the proximal LAD. The table shows the regression coefficients (B), standard errors (SE), Wald statistics, *p* values, odds ratios (Exp(B)), and 95% confidence intervals (CI) for the odds ratios of each variable.

Variable	B	SE	Wald	*p* Value	Exp (B)	95% LCI	95% UCI
LMBA > 80°	1.672	0.258	42.08	<0.001	5.325	4.029	6.701
High-risk Plaque	0.752	0.435	2.99	<0.01	2.120	1.385	3.246
CAC Score > 180	0.039	0.015	6.74	0.01	1.04	1.01	1.07
Obstructive CAD	0.788	0.274	8.23	0.02	2.20	1.40	3.50
Diabetes	1.321	0.269	24.18	<0.001	3.745	2.691	4.713

## Data Availability

All data not included in the manuscript are available by contacting the corresponding author in accordance with local legal regulations.

## Supplementary Materials

The following supporting information can be downloaded at: https://www.mdpi.com/article/10.3390/jcdd11110338/s1, Figure S1: distribution of LMBA angles across the study population. Figure S2: Comparison between the receiver operating characteristics (ROC) curves for the LMBA in the prediction of LAD stenosis. Figure S3: Comparison between the receiver operating characteristics (ROC) curves for the CLAP score. Figure S4: Receiver Operating Characteristic (ROC) curve for the CLAP score to predict disease progression in patients with follow-up imaging. Figure S5: Receiver Operating Characteristic (ROC) curves comparing the predictive accuracy of the models with and without patients with prior PCI. Table S1: Comparison of clinical outcomes between patients with LMBA > 80° and LMBA ≤ 80°. Table S2: Logistic regression analysis for predictors of clinical and anatomical predictors of proximal LAD stenosis. Table S3: CLAP Score: Variables, Points, and Risk Categories. Table S4: Risk Categories and Probabilities Based on CLAP Score. Table S5: Baseline Characteristics of Adjusted Population, excluding patients with prior PCI of the LAD. Table S6: Cox Proportional Hazards Model for MACE in Adjusted Population, excluding patients with prior PCI of the LAD.
