# Supplementary material for: Comprehensive Risk Assessment of LAD Disease Progression in CCTA: The CLAP Score Study
Source: J Cardiovasc Dev Dis. 2024 Oct 23;11(11):338. doi: 10.3390/jcdd11110338 (PMC11595042; doi:10.3390/jcdd11110338)
Supplement: Supplementary file 1 [file jcdd-11-00338-s001.zip › jcdd-3211311-supplementary.pdf]

Supplemetary Figures and Tables

Figure S1. Histogram showing the distribution of LMBA angles across the study population.

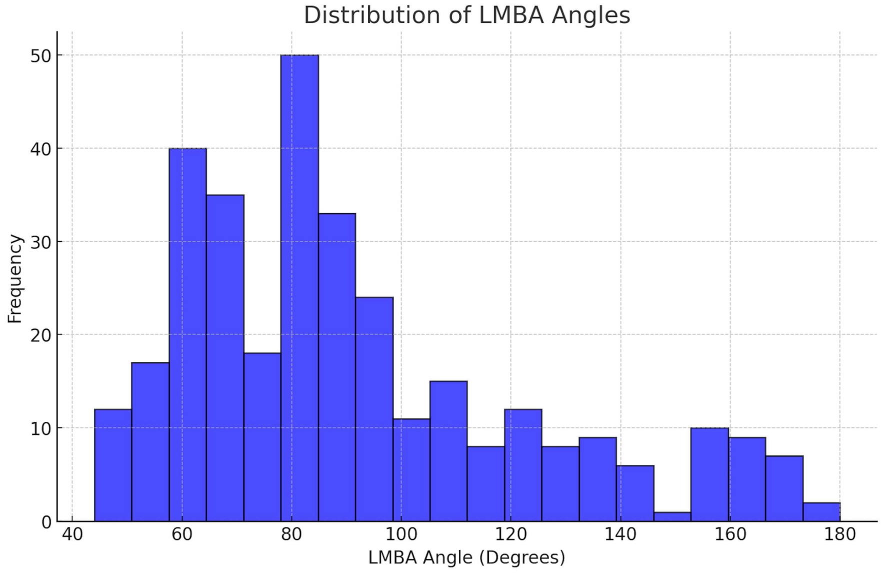

Figure S2. Comparison between the receiver operating characteristics (ROC) curves for the LMBA in the prediction of LAD stenosis. The area under the curve (AUC) is 0.84 for CCTA and 0.86 for 3D-QCA. The difference in AUC values between the modalities was -0.018 (SE: 0.0046, 95% CI: -0.0271 to -0.0090, p = 0.00009

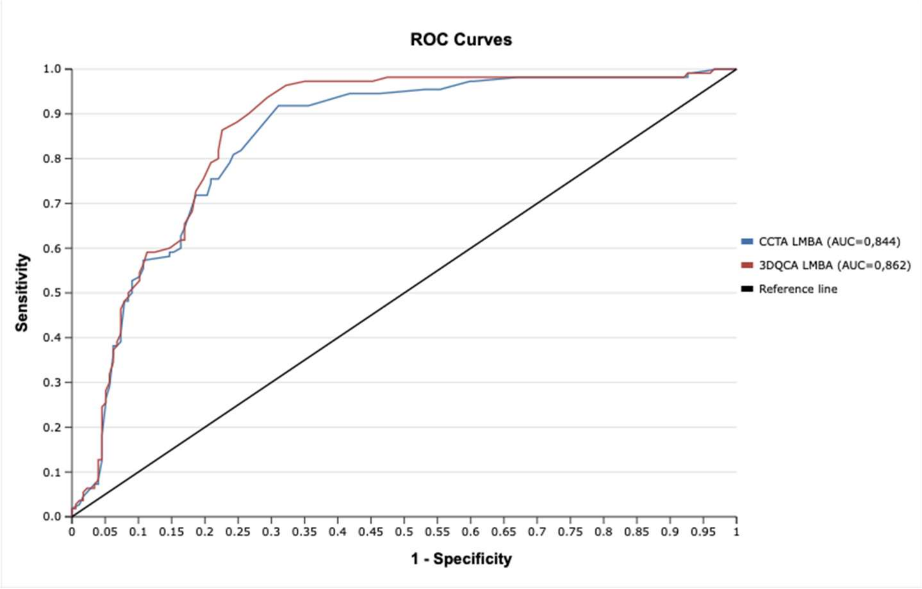

Figure S3. Comparison between the receiver operating characteristics (ROC) curves for the CLAP score. The area under the curve (AUC) demonstrated a good discriminatory ability in both the development cohort (AUC 0.91; 95% CI: 0.86-0.96) and the external validation cohort (AUC 0.85; 95% CI: 0.79-0.91), $p < 0.001$.

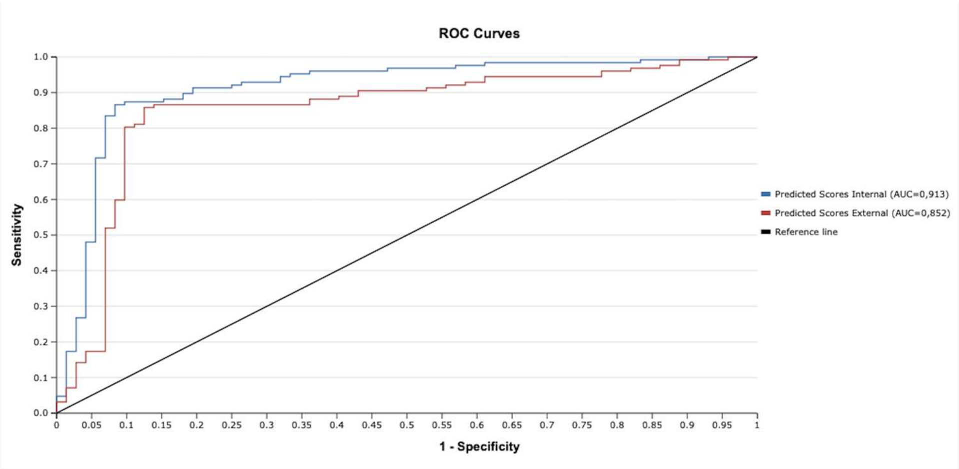

Figure S4. The Receiver Operating Characteristic (ROC) curve for the CLAP score to predict disease progression in patients with follow-up imaging. The area under the curve (AUC) is 0.89, indicating a good discriminatory ability of the CLAP score between patients with and without significant disease progression. The curve shows a balance between sensitivity and specificity, with improved predictive accuracy

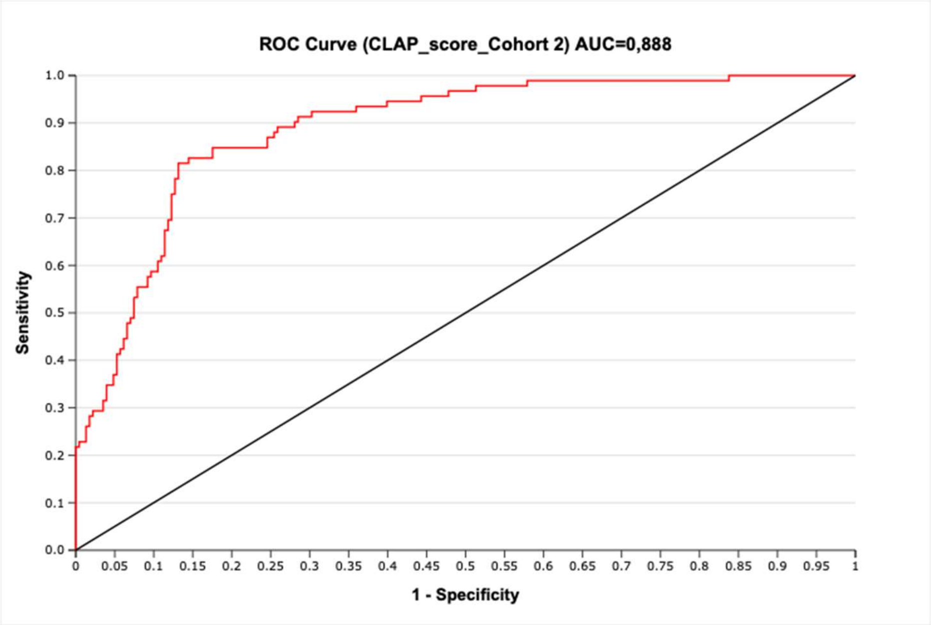

Table S1.

Outcome	Group 1 (LMBA > 80°, n = 202)	Group 2 (LMBA ≤ 80°, n = 297)	p-value
Total MACE	40	22	0.016
Cardiovascular Death	2	1	0.45
Myocardial Infarction	4	1	0.09

PCI	25	10	0.002
TLR	9	3	0.04
Progression of Disease in Proximal LAD	30	12	0.007

Comparison of clinical outcomes between patients with LMBA > 80° and LMBA ≤ 80°. Major adverse cardiovascular events (MACE), percutaneous coronary intervention (PCI), target lesion revascularization (TLR)

Table S2.

Variable	B	SE	Wald	p value	Exp(B)	95% LCI	95% UCI
Hypertension	1.228	0.285	5.012	0.025	1.228	1.102	2.024
Diabetes	2.006	0.274	53.456	<0.001	7.437	4.343	12.734
Renal Failure	0.705	0.323	4.762	0.029	2.024	1.074	3.811
LMBA >80° (CCTA)	1.169	0.243	22.998	<0.001	3.234	2.025	4.442
High Tortuosity	1.083	0.356	9.252	0.002	2.954	1.47	5.935
CAC Score (≥ 180)	0.545	0.194	7.897	0.005	1.725	1.195	2.490
Obstructive CAD	1.029	0.320	10.356	0.001	2.80	1.800	4.200
High-risk Plaque Features	0.896	0.258	12.079	<0.001	2.45	1.50	4.01
Female Sex	-0.087	0.231	0.141	0.707	0.917	0.583	1.443
Smoke	0.099	0.315	0.1	0.752	1.104	0.596	2.046
Age	-0.001	0.013	0.011	0.918	0.999	0.973	1.025
Family History of CAD	0.086	0.263	0.106	0.745	1.089	0.651	1.824
Dyslipidemia	0.482	0.261	3.399	0.065	1.619	0.97	2.701
Constant	-4.303	1	18.501	<.001	0.014		

Logistic regression analysis for predictors of clinical and anatomical predictors of proximal LAD stenosis. The table shows the regression coefficients (B), standard errors

(SE), Wald statistics, p values, odds ratios (Exp(B)), and 95% confidence intervals (CI) for the odds ratios of each variable.

Table S3

Variable	Value	Points
LMBA > 80°	Yes	3
Diabetes	Yes	2
Obstructive CAD	Yes	2
High-risk Plaque	Yes	2
CAC Score > 180	Yes	1

This table summarizes the variables included in the CLAP score, the points assigned to each variable, and the risk categories based on the total CLAP score.

Table S4

Risk Category	Total Points	Probability of Event Within One Year
Low	0-2	5% - 15%
Intermediate	3-6	40% - 55%
High	6-10	85%-95%

Risk Categories and Probabilities Based on CLAP Score This table categorizes patients into risk groups based on their total CLAP score and indicates the probability of an event occurring within one year for each risk category.

Sensitivity Analysis

Population Adjusted for Sensitivity Analysis

From the initial cohort of 499 patients, 110 patients (22.0%) with prior PCI of the LAD were excluded. This left us with a total of 389 patients for the sensitivity analysis.

Table S5

Variable	Sensitivity Population (n=389)
Age (yrs)	63.9 ± 9.8
Male	302 (77.6%)
Hypertension	282 (72.5%)
Dyslipidemia	218 (56.0%)
Diabetes Mellitus	87 (22.4%)
Renal Failure	75 (19.3%)
Significant Proximal LAD Disease	140 (36.0%)

Baseline Characteristics of Adjusted Population

After excluding patients with prior PCI, the Cox proportional hazards model was updated to determine if there were any significant changes in predictors of MACE:

Table S6

Variable	HR Sensitivity Analysis (95% CI), p-value
Diabetes	HR =2.85; 95% CI: 1.40 - 4.32, p = 0.035
Renal Failure	HR = 1.65; 95% CI: 1.25 - 5.92, p = 0.048

High-risk Plaque Morphology	HR = 2.20; 95% CI: 1.31 - 3.72, p = 0.007
LMBA > 80°	HR = 4.22; 95% CI: 3.50 - 6.20, p < 0.001
Obstructive CAD	HR = 2.30; 95% CI: 1.35 - 3.90, p = 0.02
CAC Score	HR = 1.03; 95% CI: 1.01 - 1.06, p = 0.006

Cox Proportional Hazards Model for MACE in Adjusted Population

The hazard ratios for the most important predictors remained generally consistent between the analyses with and without prior PCI patients. LMBA > 80° continued to be the strongest predictor of MACE in both models, with only a slight reduction in the hazard ratio from 4.47 to 4.22. The AUC for the model without patients with prior PCI was 0.857 (95% CI: 0.798 - 0.916), compared with 0.896 (95% CI: 0.841 - 0.952) in the overall population (Fig 4A)

Figure S5: Receiver Operating Characteristic (ROC) curves comparing the predictive accuracy of the models with and without patients with prior PCI. The blue curve represents the model including all patients AUC = 0.896, 95% CI: 0.841 - 0.952), while the red curve represents the model excluding patients with prior PCI (AUC = 0.857, 95% CI: 0.798 - 0.916). Despite the exclusion of prior PCI patients, the model retained strong predictive value with only a slight reduction in AUC.

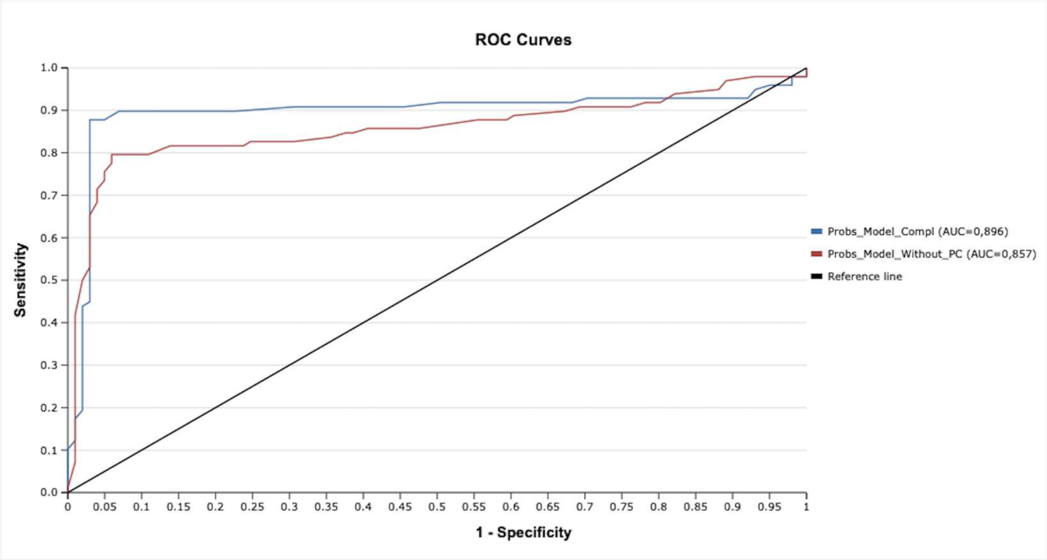

.

The revised CLAP score maintained similar predictive accuracy, with an AUC of 0.88 (95% CI: 0.82 - 0.92) for the development cohort and 0.83 (95% CI: 0.78 - 0.88) for the external validation cohort. The risk categories continued to be effective in stratifying patients into low, intermediate and high risk groups, with similar probabilities of disease progression across categories. Exclusion of patients with prior PCI did not significantly alter the predictors of MACE. LMBA > 80°, high-risk plaque morphology and diabetes remained strong predictors of disease progression in the matched population. The CLAP score maintained its ability to effectively stratify risk even without the inclusion of patients with prior PCI. These results suggest that although the inclusion of patients with prior PCI represents a higher-risk population, it did not substantially bias the overall predictive results of our model or affect the robustness of the CLAP score.
